# Role of Mitochondrial Dysfunction in Degenerative Brain Diseases, an Overview

**DOI:** 10.3390/brainsci8100178

**Published:** 2018-09-20

**Authors:** Ruben K. Dagda

**Affiliations:** Department of Pharmacology, Reno School of Medicine, University of Nevada, 1664 North Virginia Street, Mail Stop 318, Reno, NV 89557, USA; rdagda@med.unr.edu; Tel.: +1-775-784-4121

## 1. Mitochondria as Major Therapeutic Targets for Neurodegenerative Diseases

Mitochondria are multifaceted organelles that serve to power critical neuronal functions. Additionally, mitochondria buffer calcium, produce intracellular reactive oxygen species (ROS), act as heat generators, regulate lipid metabolism, and modulate cell survival.

Unlike proliferating cells, neurons predominantly rely on oxidative phosphorylation to thrive and establish neuronal networks. Although the brain comprises less than 2% of human body weight, it consumes of up to 20% of the total energy levels produced by the body. Indeed, the high reliance of neurons on oxidative phosphorylation is evident as mitochondrial dysfunction underlies the etiology of a many neurodegenerative disorders including Alzheimer’s disease (AD), Amyotrophic Lateral Sclerosis (ALS), Huntington’s disease (HD), optic neuropathy (ON), Marie-Charcot-Tooth (MCT) disease, and Parkinson’s disease (PD) [1,2,3,4]. In addition, several studies using cell culture and in vivo models have provided convincing proof-of-concept that high quality/functional mitochondria are critical for neuronal survival. For instance, mutations in several proteins that regulate mitochondrial function and structure (e.g., PINK1) give rise to early onset familial forms of PD that are characterized by the degeneration of the midbrain and cortex, neuropathology that is associated with the presence of a variety of non-motor symptoms (e.g., sleep disorders and constipation) [5,6]. Furthermore, mitochondrial dysfunction, impaired mitochondrial trafficking, and mitochondrial fragmentation is recapitulated in neuronal cell culture models that express PD-associated mutants of PTEN-induced kinase 1 (PINK1), LRRK2, and DJ-1 [7,8,9]. Interestingly, loss of PINK1 function has been shown to contribute to neuropathology and cognitive decline in an in vivo model of AD [10], raising the possibility that PD-associated genes, or other brain degenerative-associated genes, contribute to disease progression in other neurodegenerative disorders. Overall, these studies suggest that mitochondrial dysfunction, caused by pathogenic mutations in specific nuclear-encoded mitochondrial proteins, is sufficient to cause neurodegeneration. 

Consistent with this concept, a mouse model that expresses a dysfunctional mitochondrial transcription factor (TFAM) (Mito-Park) faithfully recapitulates several aspects of PD pathology, including a significant loss of midbrain dopamine neurons, deficits in neurotransmitter levels, and loss of motor movement [11]. In neurodegenerative diseases, it is important to bear in mind that damaged mitochondria are not only characterized as unable to produce a sufficient level of ATP via oxidative phosphorylation, but are also impaired for movement (trafficking), incapable to undergo efficient fission or fusion, mitochondrial turnover (mitophagy) or be replaced via mitochondrial biogenesis. Each of these features of mitochondrial dysfunction is further elaborated below. Therefore, elevating mitochondrial function is a viable therapeutic strategy for reversing neurodegeneration in neurodegenerative diseases associated with mitochondrial dysfunction.

## 2. Role of Mitochondrial Fission/Fusion in Brain Health and in Neurodegenerative Diseases

Mitochondria are highly dynamic organelles that exhibit bidirectional mobility and undergo mitochondrial fission (fragmentation) or fusion events (tubular to interconnected network). Mitochondrial fission and fusion (MFF) is a physiological process modulated by large mitochondrial GTPases of the dynamin super family, localized at the outer mitochondrial membrane (OMM) or the inner mitochondrial membrane (IMM). MFF regulators can sculpt mitochondria into spherical, tubular, or highly interconnected networks. Mitochondrial fission, as regulated by the association of cytosolic localized dynamin related protein 1 (Drp1) with Fis1 at the OMM, is an early molecular event that is required for eliciting apoptosis. On the other hand, blocking fission can halt or delay apoptosis in a variety of cell types, including neurons [12,13]. The extent of MFF activity can govern critical cellular functions including cellular bioenergetics, Ca^2+^ buffering, free radical production, mitochondrial content, cell survival, mitochondrial DNA inheritance, and mitophagy [13,14]. Conversely, upregulating the level of mitochondrial fusion regulators (e.g., increased Opa1 and Mfn2) can reverse mitochondrial fragmentation induced by NMDA-mediated excitotoxicity, thereby blocking neuronal death [15].

In addition to preserving the integrity of the soma, emerging evidence suggests that a minimum amount of functional mitochondria is critical for the maintenance of neuronal functions including the development of dendrites and dendritic spines, the release of neurotransmitters, the induction of long-term potentiation, and mobilizing the reserve pool of neurotransmitter vesicles at the synapse [1,14,16,17]. 

## 3. Role of Mitochondrial Content in Brain Health and in Neurodegenerative Diseases

There are several factors that control the number of mitochondria present in dendrites (mitochondrial content), axons, and synaptic terminals: mitochondrial dynamics, the level of mitochondrial trafficking, the level of mitochondrial turnover, the activation of mitochondrial biogenesis programs, and the level of oxidative stress [18]. 

Mitochondrial trafficking is governed by well-characterized molecular complexes localized at the OMM that include ATP-dependent dynein and kinesin motors, mitochondrial-localized GTPases (Miro1 and 2), and mitochondrial-specific motor adaptor proteins (TRAK1 and 2, orthologs of Milton) [18,19]. In the axons, while kinesins induce anterograde trafficking of cargo (away from soma), dynein motors move cargo in a retrograde manner (toward the soma) [20]. Large mitochondrial adaptor proteins such as Rho GTPase proteins (Miro1/2) regulate the movement of mitochondria, maintain mitochondrial integrity [21,22], and regulate mitochondrial trafficking in dendrites in vivo [21].

Proper mitochondrial trafficking activity within neurites (dendrites/axons) is critical for neuronal survival, proper dendrite development, the formation of dendritic spines, and/or proper neuronal connectivity in the brain. Indeed, deficits in mitochondrial trafficking have been proposed to contribute to the pathogenesis of PD, ALS, ON, CMT, and AD disease [16,23,24,25]. In addition, some neurodegenerative diseases are characterized by a dysfunction of the Miro/Milton complexes and of kinesin and dynein motors, which leads to a complete cessation of mitochondrial trafficking and mitochondrial dysfunction in dendrites and axons [16,26]. Therefore, a minimum level of mitochondrial trafficking allows mitochondria to move towards sites of highest energy demands within dendritic arbors and axons to help maintain neuronal networks and promote neuronal survival [18,27].

## 4. Role of Mitophagy in Brain Health and in Neurodegenerative Diseases

Oxidatively damaged mitochondria are continuously targeted for autophagolysosomal-mediated degradation via a selective physiological process termed mitophagy [28,29,30]. While macroautophagy can non-selectively degrade large protein aggregates and other cytosolic cargo/organelles, mitophagy is a highly specific, well-characterized process in mammalian cells. Mitophagy is intrinsically governed by a variety of cytosolic and mitochondrial intermediate players (e.g., PINK1 and Parkin) and downstream “effectors” (e.g., LC3 and P62) [29,30,31,32]. Adding another layer of complexity on regulation of mitophagy, the MFF machinery interplays with the autophagic machinery to modulate mitophagy [33]. Consistent with this concept, an increase in Drp1-dependent mitochondrial fission [33], activated with a low level of oxidative stress, facilitates the removal of mitochondria via the autophagolysosomal pathway [34,35]. Hence, an increase in the turnover of mitochondria not only allows for the removal of damaged/effete mitochondria, but additionally allows for the concomitant activation of mitochondrial biogenesis pathways, critical for maintaining a proper level of functional mitochondria in neurons [32]. On the other hand, either overactive or impaired mitophagy within dendrites or axons can contribute to the early loss of neurites (axons/dendrites) in neurodegenerative diseases [8,36,37,38].

To date, several types of mitophagy have been characterized in eukaryotic cells. In neurons, mitophagy involves the interaction of two PD-associated enzymes: the mitochondrial ser/thr kinase PINK1 and the E3 ubiquitin ligase Parkin (PARK2) [30]. Indeed, treating neuronal cells with mitochondrial depolarizing agents such as carbonyl cyanide 4-(trifluoromethoxy) phenylhydrazone (CCCP) or valinomycin lead to a collapse of the mitochondrial membrane potential followed by a rapid accumulation of PINK1 to the OMM. The loss of mitochondrial transmembrane potential in neurons induces a rapid translocation of Parkin (a PINK1 substrate) from the cytosol to the mitochondria. At the OMM, PINK1 activates Parkin through phosphorylation of ubiquitin, which then tags OMM-localized proteins (e.g., VDAC) for ubiquitin-proteasome-mediated degradation, and thereby, facilitates the rapid mitochondrial translocation and interaction of the autophagy receptor LC3 with P62, a protein adaptor that links both ubiquitin-proteasome and mitophagy pathways [39,40,41,42]. Recently, additional molecular players that regulate mitophagy in neurons have been discovered including Optineurin and other mitophagy modulators, which have been shown to interact with PD, ALS, and HD-associated proteins [43,44]. Overall, these published observations warrant future research to analyze how MFF and mitophagy regulators interplay with other brain degenerative disease-linked proteins to govern mitochondrial structure/function and mitophagy.

A second form of mitochondrial quality control pathway involves the formation of mitochondrial-derived vesicles (MDVs), a phenomenon that is tightly controlled by PINK1 and Parkin. The mitochondrial cargo (predominantly oxidative-damaged complex proteins) contained in MDVs is specifically targeted for degradation by lysosomes [45]. Importantly, there is proof-of-concept that MDVs can be formed in the brain in vivo [46]. However, while it is widely accepted that PINK1/Parkin/ubiquitin-mediated mitophagy regulates the bulk of quality control of mitochondria, it is conceivable that mitochondrial dysfunction induced by oxidative stress in neurons affected with neurodegenerative diseases may impair the induction of the formation and trafficking of MDVs to the lysosomes, and thereby, adversely affecting mitochondrial quality control.

Mitochondrial biogenesis is a multistep process that involves the coordinated transcription/translation of mitochondrial-encoded proteins, membrane biogenesis, and assembly of outer mitochondrial membranes, which ultimately forms a mitochondrion de novo. Hence, mitophagy and mitochondrial biogenesis represent opposing physiological responses that tightly regulate levels of mitochondria in the soma and dendrites/axons of neurons. Indeed, an increase in mitophagy not only allows for the removal of damaged mitochondria but also for the coordinated induction and integration of mitochondrial biogenesis pathways to facilitate the recuperation of functional mitochondria [32]. 

Mitochondrial biogenesis is tightly regulated with the interplay of several molecular players, including cAMP response element binding protein (CREB), the transcription nuclear respiratory factor 1 (NRF1), transcription factor activator A, mitochondrial (TFAM), and PPARG Coactivator 1 Alpha (PPARGC1A) [47,48]. Recent evidence suggest that oxidative stress induced by neurodegenerative diseases adversely affects mitochondrial biogenesis pathways. Alterations in the level and activity of the transcription factor CREB, which regulates expression of PPARGC1A, contributes to impaired mitochondrial biogenesis in PD, AD, and HD models [49,50,51,52]. Furthermore, decreased expression of PPARGC1A has been observed in different in vivo and in vitro models of AD, HD, and PD postmortem brain tissue [51,53]. In addition to the PKA-CREB signaling axis, PINK1 and Parkin regulate mitochondrial biogenesis as PINK1 and Parkin-deficient cells show impaired mitochondrial biogenesis [54,55], whereas overexpression of Parkin has the opposite effect [56]. Therefore, an imbalance in either induction of biogenesis or mitophagy in neurons, as induced by oxidative stress or pathological stimuli, can lead to decreased mitochondrial content in the soma and in neuronal connections, and thereby, contribute to early stages of neurodegeneration in a variety of brain degenerative diseases. However, the pathological mechanisms that lead to the impairment of signaling pathways that govern mitochondrial structure/function, trafficking, biogenesis, and quality control need to be functionally dissected in in vitro and in vivo models of neurodegenerative diseases. Therefore, increasing our understanding on the pathological mechanisms that impair mitochondrial function in neurons will be critical for developing new drugs that can restore mitochondrial health, and thereby, halt neurodegeneration in neurodegenerative brain diseases.

## 5. Invitation to Submit Original Research Manuscript and Reviews for This Special Issue

As a guest editor for this special *Brain Sciences* issue of “Pathological Mechanisms of Neurodegenerative Diseases Associated with Impairments in Mitochondrial Function and Quality Control”, it is a pleasure to welcome the submission of original research manuscripts and reviews that shed light on the molecular mechanisms that contribute to the progression of neurodegenerative diseases associated with mitochondrial dysfunction. Primary research papers and reviews may involve scholarly work that offers novel insights into pathobiological mechanisms of brain degenerative diseases associated with mitochondrial dysfunction, at the basic science, preclinical, and clinical level. It is my hope that the breadth of knowledge presented in this special issue will advance our understanding on neuropathological mechanisms in neurodegenerative diseases, as well as offering insights into new “mito-protective therapies” available in the pipeline to treat brain degenerative disorders.

## References

[1] Dagda R.K., Das Banerjee T. (2015). Role of protein kinase A in regulating mitochondrial function and neuronal development: Implications to neurodegenerative diseases. Rev. Neurosci..

[2] Gusdon A.M., Chu C.T. (2010). To eat or not to eat: Neuronal metabolism, mitophagy, and Parkinson’s disease. Antioxid. Redox Signal..

[3] Ayala A., Venero J.L., Cano J., Machado A. (2007). Mitochondrial toxins and neurodegenerative diseases. Front. Biosci..

[4] Beal M.F. (1995). Aging, energy, and oxidative stress in neurodegenerative diseases. Ann. Neurol..

[5] Dagda R.K., Zhu J., Chu C.T. (2009). Mitochondrial kinases in Parkinson’s disease: Converging insights from neurotoxin and genetic models. Mitochondrion.

[6] Dauer W., Przedborski S. (2003). Parkinson’s disease: Mechanisms and models. Neuron.

[7] Dagda R.K., Gusdon A.M., Pien I., Strack S., Green S., Li C., Van Houten B., Cherra S.J., Chu C.T. (2011). Mitochondrially localized PKA reverses mitochondrial pathology and dysfunction in a cellular model of Parkinson’s disease. Cell Death Differ..

[8] Cherra S.J., Steer E., Gusdon A.M., Kiselyov K., Chu C.T. (2013). Mutant LRRK2 elicits calcium imbalance and depletion of dendritic mitochondria in neurons. Am. J. Pathol..

[9] Bonifati V., Rizzu P., Squitieri F., Krieger E., Vanacore N., van Swieten J.C., Brice A., van Duijn C.M., Oostra B., Meco G. (2003). DJ-1(PARK7), a novel gene for autosomal recessive, early onset parkinsonism. Neurol. Sci..

[10] Du F., Yu Q., Yan S., Hu G., Lue L.F., Walker D.G., Wu L., Yan S.F., Tieu K., Yan S.S.D. (2017). PINK1 signalling rescues amyloid pathology and mitochondrial dysfunction in Alzheimer’s disease. Brain.

[11] Galter D., Pernold K., Yoshitake T., Lindqvist E., Hoffer B., Kehr J., Larsson N.G., Olson L. (2010). MitoPark mice mirror the slow progression of key symptoms and L-DOPA response in Parkinson’s disease. Genes Brain Behav..

[12] Wasilewski M., Scorrano L. (2009). The changing shape of mitochondrial apoptosis. Trends Endocrinol. Metab..

[13] Cheung E.C., McBride H.M., Slack R.S. (2007). Mitochondrial dynamics in the regulation of neuronal cell death. Apoptosis.

[14] Princz A., Kounakis K., Tavernarakis N. (2018). Mitochondrial contributions to neuronal development and function. Biol. Chem..

[15] Jahani-Asl A., Pilon-Larose K., Xu W., MacLaurin J.G., Park D.S., McBride H.M., Slack R.S. (2011). The mitochondrial inner membrane GTPase, optic atrophy 1 (Opa1), restores mitochondrial morphology and promotes neuronal survival following excitotoxicity. J. Biol. Chem..

[16] Das Banerjee T., Dagda R.Y., Dagda M., Chu C.T., Rice M., Vazquez-Mayorga E., Dagda R.K. (2017). PINK1 regulates mitochondrial trafficking in dendrites of cortical neurons through mitochondrial PKA. J. Neurochem..

[17] Li Z., Okamoto K., Hayashi Y., Sheng M. (2004). The importance of dendritic mitochondria in the morphogenesis and plasticity of spines and synapses. Cell.

[18] Schwarz T.L. (2013). Mitochondrial Trafficking in Neurons. Cold Spring Harb. Perspect. Biol..

[19] Van Spronsen M., Mikhaylova M., Lipka J., Schlager M.A., van den Heuvel D.J., Kuijpers M., Wulf P.S., Keijzer N., Demmers J., Kapitein L.C. (2013). TRAK/Milton motor-adaptor proteins steer mitochondrial trafficking to axons and dendrites. Neuron.

[20] Zheng Y., Wildonger J., Ye B., Zhang Y., Kita A., Younger S.H., Zimmerman S., Jan L.Y., Jan Y.N. (2008). Dynein is required for polarized dendritic transport and uniform microtubule orientation in axons. Nat. Cell Biol..

[21] Babic M., Russo G.J., Wellington A.J., Sangston R.M., Gonzalez M., Zinsmaier K.E. (2015). Miro’s N-terminal GTPase domain is required for transport of mitochondria into axons and dendrites. J. Neurosci..

[22] Weihofen A., Thomas K.J., Ostaszewski B.L., Cookson M.R., Selkoe D.J. (2009). Pink1 forms a multiprotein complex with Miro and Milton, linking Pink1 function to mitochondrial trafficking. Biochemistry.

[23] Rui Y., Tiwari P., Xie Z., Zheng J.Q. (2006). Acute impairment of mitochondrial trafficking by beta-amyloid peptides in hippocampal neurons. J. Neurosci..

[24] Shirendeb U.P., Calkins M.J., Manczak M., Anekonda V., Dufour B., McBride J.L., Mao P., Reddy P.H. (2012). Mutant huntingtin’s interaction with mitochondrial protein Drp1 impairs mitochondrial biogenesis and causes defective axonal transport and synaptic degeneration in Huntington’s disease. Hum. Mol. Genet..

[25] Correia S.C., Perry G., Moreira P.I. (2016). Mitochondrial traffic jams in Alzheimer’s disease—Pinpointing the roadblocks. Biochim. Biophys. Acta.

[26] Pareyson D., Saveri P., Sagnelli A., Piscosquito G. (2015). Mitochondrial dynamics and inherited peripheral nerve diseases. Neurosci. Lett..

[27] Cherra S.J., Dagda R.K., Chu C.T. (2010). Review: Autophagy and neurodegeneration: Survival at a cost?. Neuropathol. Appl. Neurobiol..

[28] Kanki T., Klionsky D.J. (2008). Mitophagy in yeast occurs through a selective mechanism. J. Biol. Chem..

[29] Lemasters J.J. (2005). Selective mitochondrial autophagy, or mitophagy, as a targeted defense against oxidative stress, mitochondrial dysfunction, and aging. Rejuvenation Res..

[30] Youle R.J., Narendra D.P. (2011). Mechanisms of mitophagy. Nat. Rev. Mol. Cell Biol..

[31] Whitworth A.J., Pallanck L.J. (2009). The PINK1/Parkin pathway: A mitochondrial quality control system?. J. Bioenerg. Biomembr..

[32] Zhu J., Wang K.Z., Chu C.T. (2013). After the banquet: Mitochondrial biogenesis, mitophagy and cell survival. Autophagy.

[33] Twig G., Elorza A., Molina A.J., Mohamed H., Wikstrom J.D., Walzer G., Stiles L., Haigh S.E., Katz S., Las G. (2008). Fission and selective fusion govern mitochondrial segregation and elimination by autophagy. EMBO J..

[34] Dagda R.K., Cherra S.J., Kulich S.M., Tandon A., Park D., Chu C.T. (2009). Loss of PINK1 function promotes mitophagy through effects on oxidative stress and mitochondrial fission. J. Biol. Chem..

[35] Frank M., Duvezin-Caubet S., Koob S., Occhipinti A., Jagasia R., Petcherski A., Ruonala M.O., Priault M., Salin B., Reichert A.S. (2012). Mitophagy is triggered by mild oxidative stress in a mitochondrial fission dependent manner. Biochim. Biophys. Acta.

[36] Moller A., Bauer C.S., Cohen R.N., Webster C.P., De Vos K.J. (2017). Amyotrophic lateral sclerosis-associated mutant SOD1 inhibits anterograde axonal transport of mitochondria by reducing Miro1 levels. Hum. Mol. Genet..

[37] Hsieh C.H., Shaltouki A., Gonzalez A.E., Bettencourt da Cruz A., Burbulla L.F., Lawrence E.S., Schule B., Krainc D., Palmer T.D., Wang X. (2016). Functional Impairment in Miro Degradation and Mitophagy Is a Shared Feature in Familial and Sporadic Parkinson’s Disease. Cell Stem. Cell.

[38] Chu C.T., Plowey E.D., Dagda R.K., Hickey R.W., Cherra S.J., Clark R.S. (2009). Autophagy in neurite injury and neurodegeneration: In vitro and in vivo models. Methods Enzymol..

[39] Narendra D., Kane L.A., Hauser D.N., Fearnley I.M., Youle R.J. (2010). p62/SQSTM1 is required for Parkin-induced mitochondrial clustering but not mitophagy; VDAC1 is dispensable for both. Autophagy.

[40] Narendra D.P., Jin S.M., Tanaka A., Suen D.F., Gautier C.A., Shen J., Cookson M.R., Youle R.J. (2010). PINK1 is selectively stabilized on impaired mitochondria to activate Parkin. PLoS Biol..

[41] Narendra D., Tanaka A., Suen D.F., Youle R.J. (2008). Parkin is recruited selectively to impaired mitochondria and promotes their autophagy. J. Cell Biol..

[42] Kawajiri S., Saiki S., Sato S., Sato F., Hatano T., Eguchi H., Hattori N. (2010). PINK1 is recruited to mitochondria with parkin and associates with LC3 in mitophagy. FEBS Lett..

[43] Chu C.T. (2018). Mechanisms of selective autophagy and mitophagy: Implications for neurodegenerative diseases. Neurobiol. Dis..

[44] Ryan T.A., Tumbarello D.A. (2018). Optineurin: A Coordinator of Membrane-Associated Cargo Trafficking and Autophagy. Front. Immunol..

[45] McLelland G.L., Soubannier V., Chen C.X., McBride H.M., Fon E.A. (2014). Parkin and PINK1 function in a vesicular trafficking pathway regulating mitochondrial quality control. EMBO J..

[46] Vincow E.S., Merrihew G., Thomas R.E., Shulman N.J., Beyer R.P., MacCoss M.J., Pallanck L.J. (2013). The PINK1-Parkin pathway promotes both mitophagy and selective respiratory chain turnover in vivo. Proc. Natl. Acad. Sci. USA.

[47] Signorile A., Micelli L., De Rasmo D., Santeramo A., Papa F., Ficarella R., Gattoni G., Scacco S., Papa S. (2014). Regulation of the biogenesis of OXPHOS complexes in cell transition from replicating to quiescent state: Involvement of PKA and effect of hydroxytyrosol. Biochim. Biophys. Acta.

[48] Scarpulla R.C. (2002). Transcriptional activators and coactivators in the nuclear control of mitochondrial function in mammalian cells. Gene.

[49] Dagda R.K., Pien I., Wang R., Zhu J., Wang K.Z., Callio J., Banerjee T.D., Dagda R.Y., Chu C.T. (2014). Beyond the mitochondrion: Cytosolic PINK1 remodels dendrites through protein kinase A. J. Neurochem..

[50] Pacelli C., De Rasmo D., Signorile A., Grattagliano I., di Tullio G., D’Orazio A., Nico B., Comi G.P., Ronchi D., Ferranini E. (2011). Mitochondrial defect and PGC-1alpha dysfunction in parkin-associated familial Parkinson’s disease. Biochim. Biophys. Acta.

[51] Rona-Voros K., Weydt P. (2010). The role of PGC-1alpha in the pathogenesis of neurodegenerative disorders. Curr. Drug Targets.

[52] Chalovich E.M., Zhu J.H., Caltagarone J., Bowser R., Chu C.T. (2006). Functional repression of cAMP response element in 6-hydroxydopamine-treated neuronal cells. J. Biol. Chem..

[53] Qin W., Haroutunian V., Katsel P., Cardozo C.P., Ho L., Buxbaum J.D., Pasinetti G.M. (2009). PGC-1alpha expression decreases in the Alzheimer disease brain as a function of dementia. Arch. Neurol..

[54] Gegg M.E., Cooper J.M., Schapira A.H., Taanman J.W. (2009). Silencing of PINK1 expression affects mitochondrial DNA and oxidative phosphorylation in dopaminergic cells. PLoS ONE.

[55] Rothfuss O., Fischer H., Hasegawa T., Maisel M., Leitner P., Miesel F., Sharma M., Bornemann A., Berg D., Gasser T. (2009). Parkin protects mitochondrial genome integrity and supports mitochondrial DNA repair. Hum. Mol. Genet..

[56] Kuroda Y., Mitsui T., Kunishige M., Shono M., Akaike M., Azuma H., Matsumoto T. (2006). Parkin enhances mitochondrial biogenesis in proliferating cells. Hum. Mol. Genet..

